# Mosaic *PTEN* alteration in the neural crest during embryogenesis results in multiple nervous system hamartomas

**DOI:** 10.1186/s40478-019-0841-0

**Published:** 2019-12-03

**Authors:** Alice Goldenberg, Florent Marguet, Vianney Gilard, Aude-Marie Cardine, Adnan Hassani, François Doz, Sophie Radi, Stéphanie Vasseur, Jacqueline Bou, Maud Branchaud, Claude Houdayer, Stéphanie Baert-Desurmont, Annie Laquerriere, Thierry Frebourg

**Affiliations:** 1 Department of Genetics, Rouen University Hospital and Normandie Univ, UNIROUEN, Inserm U1245, Normandy Centre for Genomic and Personalized Medicine, F76000 Rouen, France; 2 Department of Pathology, Rouen University Hospital, F76000 Rouen, France; 3 Department of Neurosurgery, Rouen University Hospital, F76000 Rouen, France; 4 Department of Paediatric Oncology, Rouen University Hospital, F76000 Rouen, France; 5 Department of Radiology, Rouen University Hospital, F76000 Rouen, France; 6 Oncology Center SIREDO, Institute Curie and University Paris Descartes, F75000 Paris, France; 7 Team for Child Development, F76130 Mont Saint-Aignan, France

**Keywords:** *PTEN*, Mosaics, Hamartomas, Central nervous system, Neural crest derivatives

## Abstract

The contribution of mosaic alterations to tumors of the nervous system and to non-malignant neurological diseases has been unmasked thanks to the development of Next Generation Sequencing (NGS) technologies. We report here the case of a young patient without any remarkable familial medical history who was first referred at 7 years of age, for an autism spectrum disorder (ASD) of Asperger type, not associated with macrocephaly. The patient subsequently presented at 10 years of age with multiple nodular lesions located within the trigeminal, facial and acoustic nerve ganglia and at the L3 level. Histological examination of this latter lesion revealed a glioneuronal hamartoma, exhibiting heterogeneous PTEN immunoreactivity, astrocyte and endothelial cell nuclei expressing PTEN, but not ganglion cells. NGS performed on the hamartoma allowed the detection of a *PTEN* pathogenic variant in 30% of the reads. The presence of this variant in the DNA extracted from blood and buccal swabs in 3.5 and 11% of the NGS reads, respectively, confirmed the mosaic state of the *PTEN* variant. The anatomical distribution of the lesions suggests that the mutational event affecting *PTEN* occurred in neural crest progenitors, thus explaining the absence of macrocephaly. This report shows that mosaic alteration of *PTEN* may result in multiple central and peripheral nervous system hamartomas and that the presence of such alteration should be considered in patients with multiple nervous system masses, even in the absence of cardinal features of PTEN hamartoma tumor syndrome, especially macrocephaly.

## Introduction

Since 2009, the development of Next Generation Sequencing (NGS) technologies allowing whole exome and genome sequencing has unmasked the mutability of the human genome with an estimate of 1,58 coding variation occurring de novo *per* exome, at the pre-zygotic level [1]. The rate of de novo variations occurring at the post-zygotic level, resulting in mosaicism, and their contribution to human diseases are probably underestimated [2]. Mosaic causal alterations in central nervous system (CNS) tumors have been described in several genes such as *NF2* in meningiomas and ependymomas [3], and *TP53* in choroid plexus tumors [4, 5] and in a case of neuroblastoma [6]. Several recent studies have also pointed to the role of somatic mutations in non-malignant neurological diseases of childhood, such as malformations of cortical development, epilepsy or autism spectrum disorders [7]. Mosaic alterations of *PTEN*, corresponding either to nucleotide variations, genomic rearrangements or 10q23 microdeletions encompassing the *PTEN* locus, have already been reported in several patients exhibiting syndromic features pathognomonic of *PTEN* hamartoma tumor syndrome (PHTS), such as macrocephaly, Lhermitte-Duclos Disease, mucosal papillomatous lesions, hamartomatous polyposis and thyroid goiter [8–11]. In one patient, the father of an index case with PHTS, clinical expression was restricted to macrocephaly [8]. Germline mosaic alterations of the *PTEN* locus, associated in *trans* with inherited *PTEN* variants, have also been reported in a distinct clinical presentation corresponding to segmental overgrowth, lipomatosis, arteriovenous malformation and epidermal nevus (SOLAMEN) syndrome due to *PTEN* nullizygosity [12, 13] and for review see ref. [14]. We report herein the case of a young patient who presented with several brain and spinal cord lesions, resulting from a mosaic *PTEN* alteration restricted to discrete neural subpopulations.

## Case presentation

The patient was an 11-year-old male, without any remarkable familial medical history. He was born at term with normal growth parameters (3100 g (15.8th centile), 53 cm (91st centile), OFC (33 cm 6th centile). He was able to walk unaided at 16 months of age. Physiotherapy was performed for slight hypotonia and moderate global coordination disorder. He developed normal language skills but presented with a mild social communication disorder and a learning disability without any cognitive impairment. He was first referred to the department of genetics at 7 years of age, for an autism spectrum disorder (ASD) of Asperger type, according to the Diagnostic and Statistical Manual of Mental Disorders, fourth edition (DSM-IV). Physical examination at this age was normal; growth parameters were in the normal range and, more notably, there was no macrocephaly (+1SD). Skin examination revealed a small congenital retro-auricular hamartoma. Blood karyotype was normal and screening for fragile X syndrome and metabolic disorders was negative. At ten years of age, the patient complained of headaches and presented painful limping and lower limb asymmetry.

**Fig. 1:**
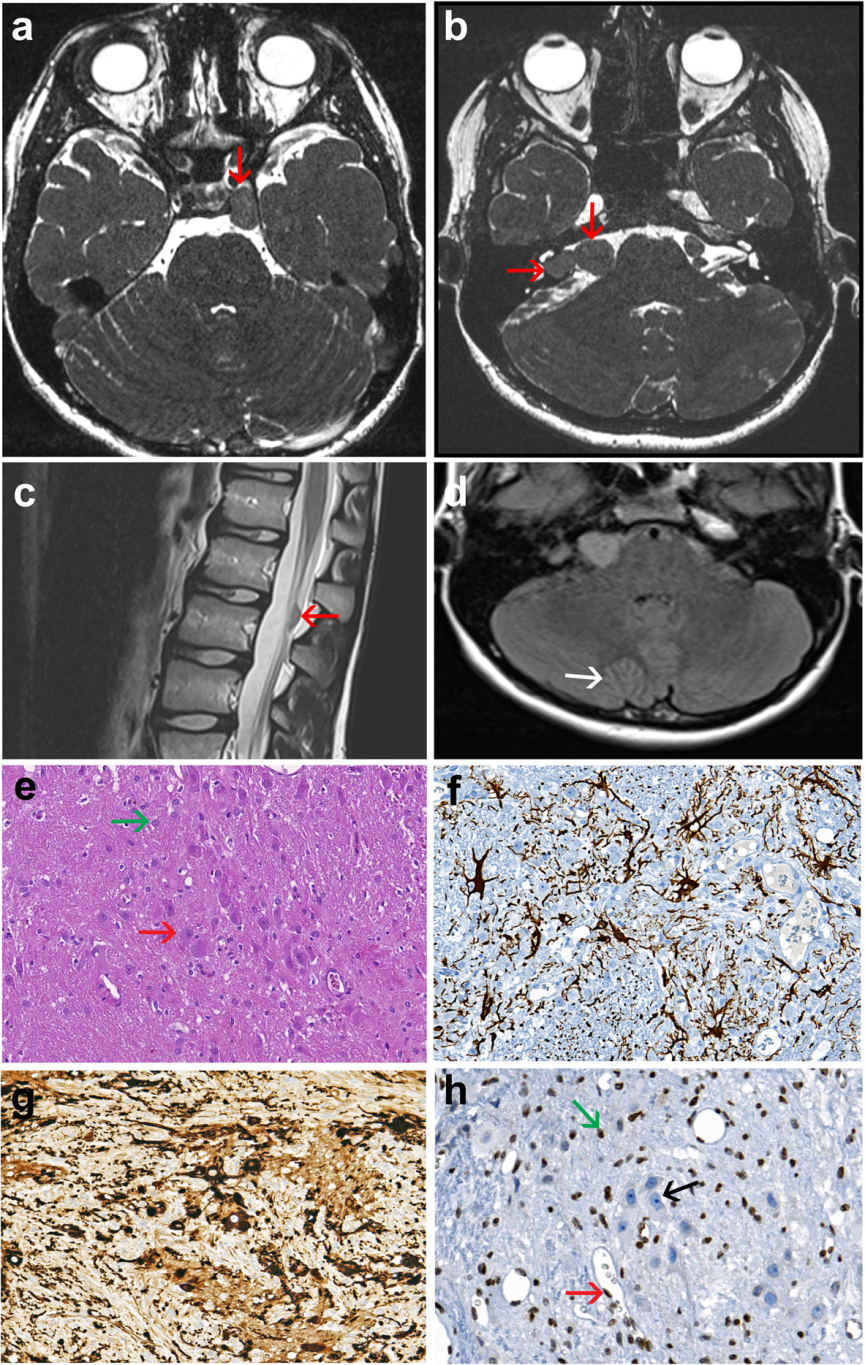
Imaging characteristics of the brain and spinal lesions; pathological hallmarks of the spinal lesion. **a-d,** MRI of the case. Axial T2-weighted images show the well circumscribed lesion located within the cavernous sinus **(a)**, at the level of the ganglion of the trigeminal nerve measuring 20 × 9 mm close to the not invaded internal carotid (red arrow) and associated with bilateral asymmetric lesions (**b**), measuring 14 × 12 mm in the interpedoncular fossa and 11 × 10 mm in the cerebellopontine angle within the ganglia of cranial nerves VII and VIII respectively (red arrows) and a nodule at the L3 level (**c**). T2 weighted axial plane of the cerebellum displaying exaggerated foliation of the right hemispheric cerebellar cortex (white arrow) located close to the vermis (**d**). **e-h,** histopathology of the resected L3 lesion. Histological view shows all but disorganized components of a spinal ganglion (**e**), including often dysmorphic ganglion cells (red arrow) and astrocytes (green arrow) lying in a fibrillar background [OM × 200]. Immunohistochemistry displays multiple GFAP positive astrocytes [OM × 200] (**f**), and numerous MAP2 positive ganglion cells [OM × 200] (**g**). PTEN immunolabeling reveals immunoreactive astrocytes (green arrow), endothelial (red arrow) and satellite cell nuclei, whereas positivity of ganglion cell (black arrow) nuclei is lost [OM × 100] (**h**). (OM: original magnification)

Magnetic resonance imaging (MRI) revealed intracranial extra-cerebral and spinal intra-dural masses, T1-hypointense, T2-hyperintense with contrast enhancement after gadolinium injection. These nodular lesions were located within the ganglion of the trigeminal, facial and acoustic nerves (Fig. 1a and b). An extramedullary intradural nodule with similar imaging characteristics was detected at the L3 level (Fig. 1c). A diagnosis of neurofibromatosis type II and schwannoma predisposition syndrome was initially considered but screening of *NF2*, *INI1*, *SMARCB1*, and *LZTR1* on the patient’s blood using NGS did not reveal any detectable germline alteration. The L3 lesion was surgically removed. Six months post-operatively, control MRI showed stable volumes of the cranial lesions. It also revealed a cerebellar cortical lesion consisting in “focal micropolygyria” of the right hemisphere (Fig. 1d), differing from Lhermitte-Duclos disease in which the cerebellar cortex appears broadened on MRI.

Histological examination of the well circumscribed L3 lesion measuring 1 cm in width revealed the presence of a heterogeneous lesion resembling a disorganized ganglion. It was composed of enlarged dysmorphic ganglion cells, either dispersed or arranged in small clusters, intermixed with protoplasmic astrocytes, spindle cells as well as small round cells lying in a fibrillar network (Fig. 1e). Neither necrosis nor mitoses were identified. Immunohistochemical study indicated that astrocytes expressed GFAP (Fig. 1f), and only scarce Olig2-positive cells were observed, indicative of defective astrocytic maturation. SOX10 and PS100 were positive in the vast majority of cells, corresponding to neural crest cells. Ganglion cells were strongly immunoreactive for MAP2 (Fig. 1g), chromogranin and NeuN. The fibrillar background was synaptophysin- and neurofilament-positive corresponding to disordered assembly of axons. CD34 immunolabelled endothelial cells only. The proliferative marker Ki67 was negative. Based on these findings, the final neuropathological diagnosis of glioneuronal hamartoma was established.

PTEN immunoreactivity appeared heterogeneous, with astrocyte and endothelial cell nuclei expressing PTEN, but not ganglion cells (Fig. 1h). To interpret the latter result, PTEN immunolabeling was performed on several adult samples including one frontal cortex sample, two sympathetic ganglia and one dorsal root ganglion, used as controls. Despite repeated immunolabelling with PTEN antibody, no immunoreactivity was detected in ganglion cells. NGS performed on DNA extracted from the L3 lesion (see Additional file 1) revealed in 30% of the reads a *PTEN* pathogenic variant within exon 8 (c.970dup; p.(Asp324Glyfs*3); NM_000314.6). No other *PTEN* alteration, corresponding either to a second pathogenic nucleotide variant or a deletion of the *PTEN* locus, was detected in the L3 lesion. This allelic imbalance was not suggestive of a germline heterozygous *PTEN* alteration, which usually yields a percentage of mutant reads close to or above 50%, when associated with loss of heterozygosity, and led us to suspect a mosaic alteration of *PTEN*. NGS performed at high depth (>500X) on DNA extracted from blood and buccal swabs found this variation in 3.5 and 11% of the reads, respectively, confirming the mosaic state of the *PTEN* variant. In order to quantify the variant allelic fraction present in the different tissues, using an independent method, we performed a targeted analysis based on QMPSF (Quantitative Multiplex PCR of Short fluorescent Fragments) and dye-labeled primers specific to *PTEN* exon 8. This analysis confirmed the presence of the *PTEN* variant in 28, 6 and 12% of the DNA extracted from the hamartoma, blood and buccal swabs, respectively (see Additional file 1). Although we could not formally exclude the fact that the detection of the *PTEN* variant in a small fraction of blood DNA corresponded to circulating DNA originating from the hamartoma, its detection in buccal swabs makes this hypothesis unlikely. Therefore the final diagnosis was a glioneuronal hamartoma resulting from a mosaic *PTEN* alteration and we extrapolated that the brain lesions also corresponded to glioneuronal hamartoma, as they were located within the Gasser, as well as within Corti and Scarpa ganglia.

## Discussion and conclusions

The PTEN protein is a phosphatase functioning as a key negative regulator of the PI3/AKT/mTOR cascade. Germline alterations of *PTEN*, cause PHTS, encompassing Cowden syndrome (CS; OMIM 158350), Bannayan-Riley-Ruvalcaba syndrome (BRRS; OMIM 153480) and Proteus and Proteus-like syndrome [for review see ref. [15, 16]. Marked macrocephaly (usually > + 3SD to +6SD) in young children, is very specific to PHTS, when compared to other syndromes with ASD, and is a required major criterion for the diagnosis of PTHS [15–17]. In Cowden mouse models, it has been shown that macrocephaly results from the increased proliferation and the decreased apoptosis of neural stem cells in the ventricular zones [18]. The absence of significant macrocephaly in our patient did not lead us to consider a diagnosis of PTHS and strongly suggested that radial glial cells and intermediate progenitors did not harbor the deleterious *PTEN* variant at embryonic stages. Despite the absence of macrocephaly, we nevertheless postulate that the Asperger syndrome or mild ASD present in this patient was probably also related to the mosaic *PTEN* variant.

As regards the development of glioneuronal hamartomas in the cranial and spinal ganglia, the mutational event in our patient very likely occurred at the end of the third post-conception week (day 18- day 21). At this time, the neural crest which arises from neuroepithelial cells adjacent to the edges of the neural groove forms two longitudinal strips on both sides of the embryonic midline and, shortly after, becomes segmented to form cranial nerve and spinal cord ganglia from the sixth post-conception week. This hypothesis is consistent with previous reports in which it has been stated that deleterious somatic mutations must occur early during development to have phenotypic effects, even though the pathogenic consequences may not be observed before childhood or adulthood [2]. It should be noticed that the NGS analysis of *PTEN* in the hamartoma did not show loss of heterozygosity. This in agreement with a previous study reporting that, in *PTEN* variation carriers, hamartomas may develop without loss of the wild-type allele [19].

There is no obvious hypothesis that could explain the detection in blood of a *PTEN* variant which occurred in neural crest progenitors. Nevertheless, recent data have revealed that neural crest cell progenitors are in fact multipotent, harbor mesenchymal potential [20] and that some hematopoietic mesenchymal stem cells derive from the neural crest [21]. The higher variant allelic fraction observed in buccal swabs, as compared to the blood, might be explained by the contribution of the neural crest to many ectomesenchymal derivatives in the cranial region.

In conclusion, this report underlines that mosaic alteration of *PTEN* is sufficient to cause multiple central and peripheral nervous system hamartomas and that the presence of such alteration should be considered in patients with multiple nervous system masses, even in the absence of cardinal features of PHTS, especially macrocephaly.

## Additional file

**Additional file 1.** Molecular assays.

## Data Availability

Most data generated or analyzed during this study are included in this article. Additional datasets are available from the corresponding author on request.

## Abbreviations

ASD: Autism spectrum disorder
CNS: Central nervous system
MRI: Magnetic resonance imaging
NGS: Next Generation Sequencing
PHTS: *PTEN* hamartoma tumor syndrome
