# Supplementary material for: Mosaic PTEN alteration in the neural crest during embryogenesis results in multiple nervous system hamartomas
Source: Acta Neuropathol Commun. 2019 Dec 3;7:191. doi: 10.1186/s40478-019-0841-0 (PMC6892231; doi:10.1186/s40478-019-0841-0)

**Molecular assays**

Sequencing of *PTEN*

*PTEN* was analysed using a NGS gene panel designed with Agilent SureDesign (Agilent, Santa Clara, California) to capture the exonic and intronic sequences plus 2 kb of the 5’ and 3’ sequences. The gene panel included *MLH1, MSH2* (plus 20 kb of 5’ sequences to capture *EPCAM*), *MSH6*, *PMS2* (only exons), *APC, MUTYH, NTHL1, MSH3, POLE, POLD1, STK11, SMAD4, BMPR1A* and *PTEN*. The total captured sequences represented 499 kb. The library preparation protocol used the SureSelect target enrichment kits from Agilent, QXT for DNA extracted from blood and buccal swabs, and XT-HS for DNA extracted from tumour, and a Sciclone NGSx workstation (PerkinElmer, Waltham, Massachusetts, USA). Libraries were sequenced on a NextSeq 500 platform (Illumina, San Diego, USA), using 2x150 bp paired-end sequencing. The panel design and the enrichment protocol are available upon request. A bioinformatic pipeline was set up to secure detection of SNVs and Indels. Reads mapping was performed using BWA and the resulting bam files were recalibrated using Picard and GATK (Broad Institute). The pipeline includes 3 variant callers (GATK Haplotype Caller, VarScan2, VarDict) and all variants detected were then annotated by SnpEff and Alamut Batch (Interactive BioSoftware, Rouen, France). A second pipeline was used for the detection of structural variants (SVs) using the CANOES and GRIDSS softwares, followed by AnnotSV for annotations. For each sequencing run, PDF quality reports integrating the number of clusters/mm2, percentage of bases with a Qscore>30, FastQC reports, percentage of mapped reads, on and off targets percentages, percentage of covered bases and mean sequencing depth, were automatically generated using the home-made tool PyQua (Python Qualitics). The percentage of positions covered with a sequencing depth >100 X was for DNA extracted from the hamartoma, blood and buccal swabs above 99,6% and the mean depth obtained for *PTEN* was 1118X, 557X and 529X, respectively.

QMPSF analysis of *PTEN*

The variant allelic fraction present in the different tissues was also evaluated using QMPSF (Quantitative Multiplex PCR of Short fluorescent Fragments).  *PTEN* exon 8 and a reference gene (*PBGD*) were PCR-amplified using dye labelled primers, and separated by electrophoresis on an ABI 3130XL sequencer. Electropherograms generated from the patient and from a control sample were superimposed, and normalized using the PBGD control fragment. The *PTEN* c.970dup variant within exon 8 generated an extra peak (see figure below).


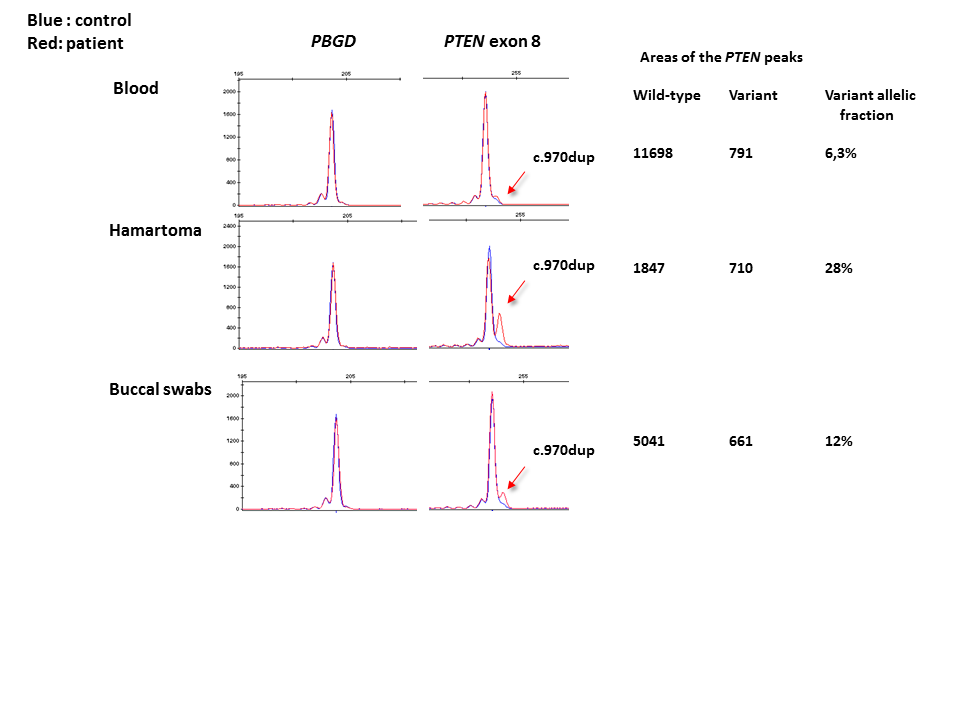

Supplement: Supplementary file 1 — Additional file 1. Molecular assays. [file 40478_2019_841_MOESM1_ESM.docx]
